# Potential Health-modulating Effects of Isoflavones and Metabolites via Activation of PPAR and AhR

**DOI:** 10.3390/nu2030241

**Published:** 2010-02-26

**Authors:** Svjetlana Medjakovic, Monika Mueller, Alois Jungbauer

**Affiliations:** 1 Department of Biotechnology, University of Natural Resources and Applied Life Sciences Vienna, Muthgasse 18, 1190 Vienna, Austria; Email: svjetlana.medjakovic@boku.ac.at (S.M.); monika.mueller@boku.ac.at (M.M.); 2 Christian-Doppler-Laboratory of Receptor Biotechnology, Muthgasse 18, 1190 Vienna, Austria

**Keywords:** isoflavones, PPARα, PPARγ, AhR, inflammation, metabolic syndrome, atherosclerosis, cell cycle control, xenobiotic metabolism

## Abstract

Isoflavones have multiple actions on cell functions. The most prominent one is the activation of estrogen receptors. Other functions are often overlooked, but are equally important and explain the beneficial health effects of isoflavones. Isoflavones are potent dual PPARα/γ agonists and exert anti-inflammatory activity, which may contribute to the prevention of metabolic syndrome, atherosclerosis and various other inflammatory diseases. Some isoflavones are potent aryl hydrocarbon receptor (AhR) agonists and induce cell cycle arrest, chemoprevention and modulate xenobiotic metabolism. This review discusses effects mediated by the activation of AhR and PPARs and casts a light on the concerted action of isoflavones.

## 1. Introduction

### 1.1. Systematics of Isoflavones

**Figure 1:**
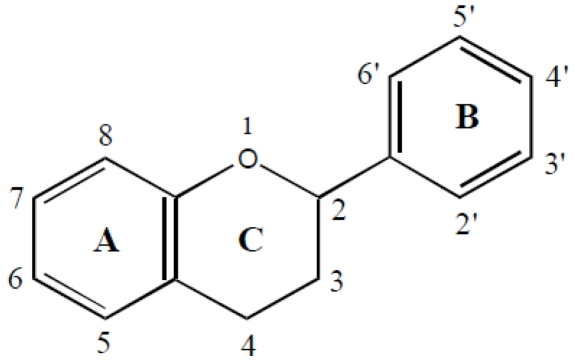
Structure of the flavonoids [with two aromatic benzol rings (A and B rings)] and a C3 portion cyclized with oxygen (C ring).

Isoflavones are a subgroup of plant phenols, which make up a group of aromatic secondary plant metabolites derived from the shikimate pathway and phenylpropanoid metabolism [1]. These compounds are widely distributed in all plant species and include simple phenol, phenolic acids, phenylacetic acids, hydroxycinnamic acids (e.g., caffeic acid, ferulic acid), coumarins, stilbens (e.g., resveratrol), flavonoids, lignans, lignins, and condensed tannins. Flavonoids are characterized by a core structure of a C6-C3-C6 flavone skeleton in which the C3 portion is commonly cyclized with oxygen (Figure 1). They vary in the degree and location of unsaturation and oxidation [1,2].

**Figure 2:**
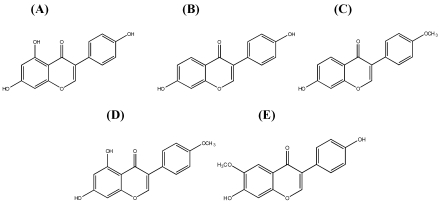
Structure of isoflavones: (A) genistein, (B) daidzein, (C) formononetin, (D) biochanin A, and (E) glycitein.

The group of flavonoids includes anthocyanins, flavans, flavanones, flavones, flavonols, and isoflavonoids. Isoflavonoids are characterized by being substituted by various hydroxyl and/or methoxy groups. This group includes, for example, genistein, daidzein, formononetin, biochanin A, and glycitein [2,3] (Figure 2).

### 1.2. Dietary Sources and Intake of Isoflavones

Isoflavones are found in trace amounts in fruits such as apples [4] and strawberries [5] and plant seeds such as sesame [5] and sunflowers [4]. But the main sources are legumes, especially the Fabaceae family, in particular soy [4,6,7] and red clover [8,9]. 

Soy is widely used in Asia as a staple food and consumed regularly in traditional food items such as tofu, miso, natto, edamame (whole soybeans), soybean paste, and shoyu (fermented soy sauce). Hence, the isoflavone intake among Asians is about a factor of 100 higher than that of people in the Western world. The daily isoflavone intake among Southeast Asians ranges between 15 and 47 mg [10,11,12,13,14,15,16], while Western people consume only between 0.15 and 1.7 mg isoflavones per day [17,18,19,20,21]. 

Red clover (*Trifolium pratense*) is widely used as a fodder crop in the Western world. In former times, it was also used in dried and milled form as a flour extender and as a salad ingredient. Today, it is mostly consumed as a food supplement for the amelioration of menopausal complaints. 

The isoflavone composition of soy and red clover differs. Soy isoflavones are mainly daidzein, genistein, and glycitein, but the predominant isoflavones of red clover are formononetin and biochanin A, while daidzein and genistein are found only in trace amounts [8,9]. 

### 1.3. Metabolism and Bioavailability of Isoflavones

Most of the isoflavones are bound as glucosides in plants. There is evidence that hydrolysis of the sugar moiety is needed for absorption [22], but the data are inconsistent; some studies report no difference between the absorption of aglycones and glucosides [23,24,25], while others found that aglycones were absorbed more efficiently [26,27]. Nevertheless, aglycone absorption seems to be unaffected by food matrix and food processing [28] or isoflavone source [29].

After oral uptake, the gastrointestinal tract is the main absorption site of isoflavones. Intestinal β-glucosidases catalyze hydrolysis of the sugar moiety  [30], and the gut microflora further metabolize the agylcones. The metabolites that result depend on the individual microflora and can differ to a great extent. During metabolism, formononetin and biochanin A are demethylated to daidzein and genistein, respectively.

The most significant metabolite, however, is certainly equol. Excretion of this metabolite of daidzein has been associated with a reduced risk of breast and prostate cancers [31,32,33,34]. The incidence of breast and prostate cancers is lower among Asians in comparison to people in the Western world [35], although breast cancer incidence is rising in Asia [36,37,38,39], probably because of lifestyle and nutrition changes that increasingly are oriented towards a Western lifestyle. Not everyone can produce equol, and the prevalence of so-called equol producers ranges from 30–50% [40,41,42,43,44,45,46,47,48,49]. 

**Figure 3:**
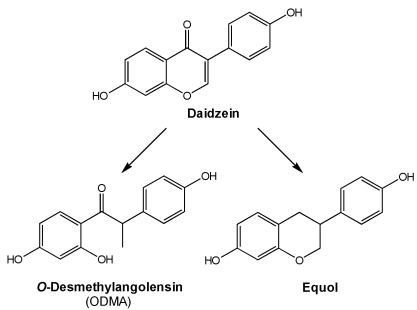
Possible metabolism products of daidzein.

Another metabolite of daidzein is *O*-desmethylangolensin (ODMA). In comparison to daidzein and equol, ODMA has a weaker affinity for estrogen receptors (ERs)  [50]. Daidzein is converted to ODMA because of a ring cleavage, while equol arises after the elimination of a carbonyl-group (Figure 3). 

Various other metabolites of isoflavones have been identified [51,52,53]. As mentioned, the emerging metabolite pattern is inter-individually different and depends on the intestinal microflora. For further information on bioavailability, there are several excellent reviews that have their main focus on this topic [54,55,56,57,58], but it should be noted that isoflavones are among the most bioavailable polyphenols.

### 1.4. Metabolic Diseases

Cardiovascular diseases like myocardial infarct and cerebrovascular diseases are the principal cause of death worldwide, representing 30% of all global deaths in 2005. If current trends continue, by 2015, an estimated 20 million people will suffer from cardiovascular diseases [59]. A sedentary lifestyle and excessive energy intake lead to an increase in the prevalence of obesity. An excess of body fat, especially visceral fat, is a key factor for developing the metabolic syndrome [60,61]. The International Diabetes Federation has defined the metabolic syndrome as central obesity (waist circumference ≥94 cm for male Europeans and ≥90 cm for male South Asians, Chinese, and Japanese and ≥80 cm for female Europeans, South Asians, Chinese, and Japanese) plus any two of the following four factors: raised triglycerides ≥150 mg/dL (1.7 mmol/L) or specific treatment for this lipid abnormality; reduced HDL (high density lipoprotein) cholesterol of <40 mg/dL (1.03 mmol/L) in males and <50 mg/dL (1.29 mmol/L) in females or specific treatment for this lipid abnormality; raised blood pressure, with a systolic blood pressure ≥130 or diastolic blood pressure ≥85 mm Hg or treatment of previously diagnosed hypertension; raised fasting plasma glucose ≥100 mg/dL (5.6 mmol/L), or previously diagnosed type 2 diabetes [62]. Cardiovascular diseases are more prevalent among patients with this syndrome [63,64,65,66,67].

Adipose tissue is an active endocrine organ producing a great variety of hormones and cytokines that are involved in glucose metabolism, lipid metabolism, inflammation, coagulation, and blood pressure. An increase in visceral fat mass is associated with an increase in secreted bioactive molecules including tumor necrosis factor (TNF)α, interleukin (IL)-6, angiotensinogen, and plasminogen activator inhibitor type 1 [68,69,70,71]. The concentration of adiponectin, a hormone that increases insulin sensitivity, has been identified to be significantly lower in the adipose tissue or serum of obese mice or humans than in lean control mice [72,73]. The enhanced secretion of inflammatory factors in adipose tissue from obese animals and humans results in a low chronic inflammatory stage that is associated with enhanced development of diabetes mellitus, the metabolic syndrome, and atherosclerosis [61,73].

#### 1.4.1. Peroxisome proliferator-activated receptors α and γ

Isoflavones activate the ligand-dependent transcription factors known as peroxisome proliferator-activated receptors (PPARs). These are class II nuclear receptors, a class that heterodimerizes with retinoid X receptor and binds to direct repeat sequences of nucleotides, which are PPAR response elements in the case of PPARs [74]. The subtypes PPARα and γ vary concerning tissue distribution. PPARγ is found mainly in adipose tissue but also in liver, kidney, intestine, and muscle [75,76]. PPARα is mainly expressed in liver, kidney, heart, muscle, and small intestine [76,77]. Furthermore, PPARγ and α are found in inflammatory and immune cells such as monocytes, macrophages, B and T cells, and dendritic cells, and in vascular wall cell types such as endothelial and smooth muscle cells, linking them to a role in inflammatory responses [76,77,78]. Fatty acids and their derivatives are the main natural ligands of all PPAR subtypes. PPARγ ligands include the fatty acids palmitic acid, petroselinic acid, oleic acid, linolenic acid, linoleic acid, and arachidonic acid [79,80], and fatty acid derivates like 15-deoxy-delta 12,14-prostaglandin J2 (15d-PGJ2) [81,82]. PPARα is activated by the peroxisome proliferator WY 14,643 and by linoleic, α-linolenic, γ-linolenic, arachidonic, docosahexaenoic, and eicosapentaenoic acids, and by eicosanoids like 8(S)-hydroxyeicosatetraenoic acid, ±8-hydroxyeicosapentaenoic acid, and carbocyclin  [81]. The synthetic ligands of PPARγ comprise the glitazones [82,83], tyrosine-based agonists, and non-steroidal anti-inflammatory drugs like fenoprofen, ibuprofen, and indomethacin  [80], and the synthetic ligands of PPARα include the fibrates  [81]. 

PPARs play a role in improving several perturbations of the metabolic syndrome. The main function of PPARγ, which has been defined as a drug target for type 2 diabetes, is adipocyte differentiation and insulin sensitization [83,84,85]. PPARγ activation leads to a modulation of factors secreted by adipose tissue. Factors that promote insulin resistance, namely TNFα, leptin, IL-6, and resistin, are reduced, and factors that promote insulin sensitivity, like adiponectin, phosphoenolpyruvate carboxykinase, fatty acid transport protein, and insulin receptor substrate-2, are upregulated [86,87,88,89,90]. Activation of PPARγ further promotes adipogenesis and lipid storage in subcutaneous adipose tissue. The result is a redistribution of adipose tissue from harmful visceral fat mass to subcutaneous depots by activation of the involved genes, including fatty acid binding protein, phosphoenolpyruvate carboxykinase, acyl-CoA synthase, diacylglycerol acyltransferase 1, fatty acid transport protein, and lipoprotein lipase [87,91,92].

PPARα activation leads to an improved lipid profile by elevating HDL levels and reducing plasma triglyceride levels. The reduction of plasma triglyceride levels is achieved by induction of genes that decrease the availability of triglycerides for hepatic very low-density lipoprotein (VLDL) secretion [93,94] and by an increased lipoprotein lipase (LPL)-mediated lipolysis of triglyceride-rich plasma lipoproteins like chylomicrons and VLDL particles [95]. This pathway is mediated by increased expression of LPL and the LPL activator apolipoprotein A-V and reduced expression of the LPL inhibitor apolipoprotein C-III [96,97]. HDL levels are elevated by increased hepatic apolipoprotein A-I and –II expression through PPARα activation [98,99].

#### 1.4.2. Inflammation and Atherosclerosis

Atherosclerosis is a complex, chronic process involving the contribution of several factors including injury to the endothelium, proliferation of vascular smooth muscle cells, migration of monocytes or macrophages, and involvement of mediators like growth factors and cytokines [100]. In brief, endothelial dysfunction, an early marker of atherosclerosis, can be induced by elevated low-density lipoproteins (LDL), hypertension, or toxins after smoking and is associated with decreased nitric oxide (NO) synthesis [101]. An inflammatory response plays a major role in the progression of atherosclerosis. Oxidized lipoprotein, T cells, and macrophages enter into the vessel wall, which leads to enhanced oxidative stress in vascular cells and to an activation of intracellular signaling molecules. T cells recognize oxidized LDL or heat shock proteins and locally release pro-inflammatory cytokines [102]. Macrophages induce collagen breakdown in atherosclerotic plaques by secreting matrix metalloproteinases (MMPs) [103,104]. In this way, the inflammatory response plays a major role in the initiation of atherosclerotic plaque formation and their destabilization. The rupture of a plaque underlies most of the acute coronary syndromes such as myocardial infarction, unstable angina, and coronary death [105].

PPARs are expressed in cells that are involved in several processes of atherosclerosis. In this way, PPARγ plays a role in improving cellular processes that contribute to atherosclerosis. Mechanisms are based on the correction of endothelial dysfunction, suppression of a chronic inflammatory process [86], reduction of foam cells and fatty streak formation [77,106], attenuating plaque evolution, and promoting plaque stabilization [107,108]. 

PPARα activation contributes to improvement of several atherosclerotic stages by downregulating pro-inflammatory genes [109] and inhibiting foam cell formation by enhancing expression of ATP-binding cassette A1 transport protein and thus increasing cholesterol efflux from macrophages and foam cells to HDL [110,111]. Furthermore, a PPARα agonist was reported to inhibit MMP-12 expression in monocyte-derived macrophages, thus leading to an inhibition of atheromatous plaque rupture [112]. By decreasing tissue factor expression, the PPARα agonist fenofibrate reduces initiation of blood coagulation and thus thrombotic complications after plaque rupture. Furthermore, fenofibrate significantly enhances endothelial regrowth and plaque stability [113]. 

#### 1.4.3. PPAR Activation in *in vitro* Assays

Activation of PPARα and γ and modulation of adipocyte differentiation in vitro are associated with putative antidiabetic or antilipidemic activity in vivo. Several studies have shown binding and/or activation of PPARα or PPARγ by the isoflavones genistein, daidzein, biochanin A, formononetin, and glycitein and the metabolites equol, ODMA, 6-hydroxydaidzein, 3´-hydroxygenistein, 6´-hydroxy-ODMA, angolensin, dihydrogenistein, dihydrobiochanin A, dihydroformononetin, dihydrodaidzein, and p-ethylphenol (Table 1). Generally, the transactivational activities were higher for biochanin A and genistein than for daidzein or formononetin. Several metabolites showed higher PPARα or PPARγ binding and activation properties than their precursors, including equol, ODMA, 6-hydroxydaidzein, and 3´hydroxygenistein [114,115].

**Table 1:** The isoflavones as PPARα and PPARγ ligands or activators.

PPARα Transactivation	PPARγ Ligands	PPARγ Transactivation	Ref
		biochanin A, genistein, daidzein, equol	[116]
	genistein	genistein	[117]
daidzein		daidzein	[118]
genistein			[119]
		daidzein	[120]
genistein, daidzein		genistein, daidzein	[121]
	biochanin A, genistein, daidzein, equol, ODMA, 6-hydroxydaidzein, 3´-hydroxygenistein, 6´-hydroxy-ODMA, angolensin, dihydrogenistein, dihydrobiochaninA, dihydroformononetin, dihydrodaidzein, p-ethylphenol	biochanin A, genistein, daidzein, equol, ODMA, 6-hydroxydaidzein, 3´-hydroxygenistein, 6´-hydroxy-ODMA, dihydrogenistein, dihydrodaidzein	[115]
biochanin A, genistein, daidzein, ODMA, 6-hydroxydaidzein, 3´-hydroxygenistein			[114]
genistein, daidzein		genistein, daidzein, glycitein	[122]
daidzein, equol			[123]
biochanin A, formononetin, genistein	biochanin A, genistein, daidzein	biochanin A, formononetin, genistein	[124]

Obesity and adipose tissue mass are associated with the number and volume of adipocytes, which result from adipocyte differentiation and triglyceride storage. Several studies have investigated the influence of isoflavones on adipocyte differentiation in 3T3-L1 cells. In these assays, 3T3-L1 preadipocytes are incubated with a differentiation medium and isoflavones simultaneously to test the effect on differentiation and the inhibition of lipid accumulation. In the maturation of preadipocytes, the transcription factors PPAR and CCAT/enhancer binding protein (C/EBPs) play a major role. First, the expression of C/EBPβ and C/EBPδ is induced by components of the differentiation medium (such as insulin, dexamethasone, and 3-isobutyl-1-methylxanthine) [125]. This induction leads to increased expression of PPAR2, C/EBPα, and sterol responsive element-binding protein (SREBP)-1, which in addition to a role in adipogenesis is responsible for the expression of mature adipocyte-specific genes like lipogenic enzymes, fatty acid binding proteins, and other secreted factors [85,126,127].

Much of the literature has focused on genistein, which inhibits adipogenesis at concentrations between 1 and 200 µM through various mechanisms: downregulation of the expression of adipocyte-specific genes including C/EBPα and β, PPARγ  [128,129], fatty acid synthase [128,129,130], adipocyte fatty acid binding protein, SREBP-1, perilipin, LPL, and hormone-sensitive lipase [128]; downregulation of the extracellular signal-regulated kinases 1 and 2 (ERK1/2) activity [131] and the action of AMP-activated kinase [132]; enhancement of leptin secretion, increased expression of the adipogenesis inhibitor preadipocyte factor 1 (Pref-1) [129], and inhibition of janus-activated kinase **(**JAK)2-mediated adipocyte differentiation [130]. Interestingly, genistein, a PPARγ activator, inhibits adipocyte differentiation *in vitro* and thereby exerts putative anti-obesity activity. Other mechanisms for putative anti-obesity activity of genistein include the inhibition of lipid accumulation in human adipocytes [128,130], possibly caused by inhibition of the activity of glycerol-3-phosphate dehydrogenase [128] and induction of apoptosis of mature adipocytes [132,133]. 

Only a few studies have investigated adipocyte differentiation in the context of the other isoflavones. Shen *et al.* [124] showed that biochanin A induces lipid accumulation in preadipocytes at a low concentration (1 µM) and formononetin and genistein at higher concentrations (3 or 15 µM). Daidzein did not induce adipocyte differentiation at this concentration range. Cho *et al.* [123] reported that daidzein enhanced adipocyte differentiation in 3T3-L1 cells at concentrations between 10 and 100 µM and C3H10T1/2 stem cells at concentrations between 1 and 20 µM and that even its metabolite equol increased adipocyte differentiation in C3H10T1/2 cells at concentrations between 0.1 and 20 µM. These data indicate the putative role of the isoflavones genistein (only at high concentrations), daidzein, formononetin, and biochanin A and the metabolite equol in fat redistribution and thus in reducing harmful visceral fat mass and simultaneously insulin resistance.

Dang *et al.* [117,118] found that in mesenchymal progenitor cells that can differentiate into osteoblasts or adipocytes, genistein and daidzein showed a biphasic effect. Adipogenesis was inhibited at low concentrations of genistein (0.1–10 µM) or daidzein (10–20 µM) and enhanced at high concentrations of genistein (>10 µM) or daidzein (>30 µM). Dang *et al.* [117,118] explained the observed effects by an interaction of PPAR and ER with activation of ER, leading to an inhibition of adipogenesis at a low concentration and PPAR activation leading to enhancement of adipogenesis at a high concentration. 

In addition to adipocyte mass, inflammation plays a major role in chronic diseases like diabetes and in the progression of atherosclerosis. Therefore, the anti-inflammatory activity of isoflavones and their metabolites in various cell culture systems is of great interest (Table 2). Cells are exposed to an inflammatory stimulus like lipopolysaccharide (LPS) or interferon (IFN)-γ. The subsequent inflammatory response is characterized by a sequential release of pro-inflammatory cytokines like TNFα, IL-6, IL-8, IL-1β, or IFN-γ  [134] The nuclear transcription factor-κB (NFκB) controls the expression of pro-inflammatory cytokines, adhesion molecules, chemokines, growth factors, or inducible enzymes such as cyclooxygenase 2 (COX-2) and the inducible nitric oxide synthase (iNOS). Successively, iNOS and COX-2 induce the production of pro-inflammatory mediators  [135]. The inflammatory state is resolved by anti-inflammatory cytokines including IL-4, IL-10, IL-13, and IFN-α [134].

In cell culture assays, isoflavones downregulate several pro-inflammatory mediators like TNFα, IL-6, IL-8, IL-1β, NO, prostaglandin E2 (PGE2), monocyte chemoattractant protein-1, IL-8, and intercellular adhesion molecule-1, or upregulate anti-inflammatory cytokines like IL-10 (Table 2). The expression of various proteins involved in the production of inflammatory mediators like iNOS, COX-2, NFκB, and signal transducer and activator of transcription 1 (STAT-1) is downregulated or their activity is inhibited. Most data on putative anti-inflammatory activity are from studies with genistein, but daidzein, formononetin, biochanin A, glycitein, and the metabolites equol and ODMA also positively influence the profile of secreted mediators.

Furthermore, isoflavones inhibit monocyte adhesion to TNFα–activated human umbilical vein endothelial cells during flow. Because monocyte adhesion to endothelial cells is among the early steps of the inflammatory cascade and contributes to atherosclerotic development, isoflavones could help to prevent atherosclerosis by this mechanism [116].

**Table 2:** Influence of isoflavones on the secretion of various inflammatory markers in cell lines.

Compounds	Cell line	Downregulated pro-inflammatory mediators	Upregulated anti-inflammatory mediators	Ref.
genistein, equol	RAW 264.7	NO, PGE2		[136]
genistein,	RAW 264.7	TNFα, IL-6, iNOS, NFκB	IL-10	[114]
daidzein,		TNFα, IL-6, iNOS, NFκB		
formononetin		iNOS		
biochanin A		TNFα, IL-6, iNOS, NFκB, Cox-2	IL-10	
equol		TNFα, IL-6, COX-2		
ODMA		TNFα, IL-6		
genistein	HBMEC	TNFα, IL-1β, monocyte chemoattractant protein-1, IL-8, intercellular adhesion molecule-1		[137]
genistein, daidzein	murine J774 macrophages	iNOS, NO		[138]
genistein	Human chondrocytes	COX-2, NO		[139]
biochanin A	MC3T3-E1 cells	TNFα, IL-6, NO		[140]
genistein	PBLs	TNFα, IL-8		[141]
genistein	mesencephalic neuron-glia cultures	TNFα, NO, superoxide		[142]
daidzein, formononetin	mesencephalic neuron-glia cultures	TNFα, NO, superoxide		[143]
biochanin A	mesencephalic neuron-glia cultures	TNFα, NO, superoxide		[144]
genistein	alveolar macrophages	TNFα		[145]
daidzein	PBMC	higher concentrations reduced IL-10 and IFN-γ levels	low concentration increased IL-2, IL-4,and IFN-γ	[146]
genistein		IL-2, IL-4, IL-10, IFN-γ mRNA and protein		
genistein	RAW 264.7	NO, PGE2		[147]
genistein	RAW 264.7	PGE2, iNOS, COX-2		[148]
genistein, daidzein, glycetein	RAW 264.7	NO, iNOS		[149]
genistein, daidzein, equol	MCF-7 cells	COX-2		[150]

HBMEC (human brain microvascular endothelial cells); MC3T3-E1 (osteoblasts); MCF-7 (human breast cancer cell line); PBL (human peripheral blood mononuclear and/or polymorphonuclear leukocytes); PBMC (peripheral blood mononuclear cells); RAW 264.7 (mouse macrophage).

#### 1.4.4. PPAR activation by isoflavones and its health effects

Given that cardiovascular diseases have reached epidemic proportions, it is of great interest that isoflavones exert *in vitro* activities that link them to putative antilipidemic, anti-obesity, antidiabetic and anti-inflammatory effects *in vivo*. The isoflavones genistein, daidzein, biochanin A, formononetin, and glycitein and several red clover metabolites like equol, ODMA, 6-hydroxydaidzein, 3´-hydroxygenistein, 6´-hydroxy-ODMA, dihydrogenistein, and dihydrodaidzein activate PPARα and γ, indicating putative antilipidemic and antidiabetic properties *in vivo*. Furthermore, adipogenesis is modulated by isoflavones. Most studies report an inhibitory effect of genistein, which may result in anti-obesity activity. Other studies report an inducing effect of genistein on adipogenesis. Biochanin A, formononetin, daidzein, and the metabolite equol enhance adipocyte differentiation and thus may promote fat redistribution from harmful visceral fat to subcutaneous fat. With a reduction in visceral fat mass, the risk for the metabolic syndrome and consequently cardiovascular diseases is reduced. Furthermore, isoflavones modulate cytokine secretion in cell culture assays, which indicates putative anti-inflammatory activities *in vivo*. Because inflammation plays a major role in atherosclerosis, anti-inflammatory activity may have a great influence on improving this disease.

Several results of *in vitro* assays are in agreement with outcomes from human or animal studies. Most animal studies were performed with genistein supplementation. An improvement of glucose levels or insulin resistance with isoflavone supplementation has been shown in obese or hypertensive rodent models [121,151,152,153] and in human studies [154]. Genistein supplementation further led to lower lipid levels and increased HDL levels [151,152,155], to an improvement in vascular health attributable to NO- and prostaglandin-dependent pathways [151,156], and to a stabilization of the atherosclerotic lesion, possibly because of reduced MMP-3 expression, based on results in rodent models and rabbits [157]. 

Supplementation with isoflavones from red clover or daidzein alone improved the lipid profile by increasing HDL and decreasing LDL, plasma total cholesterol, or triglyceride levels in rodent or rabbit models [153,158]. Furthermore, supplementation with isoflavones led to an attenuation of atherosclerosis in studies with rabbits, possibly because of an inhibition of LDL oxidation [159] or reduction of fatty streak formation [158]. 

In human studies with postmenopausal women with type 2 diabetes, isoflavones from red clover reduced diastolic and systolic blood pressure [160]. With administration of only 40 mg of isoflavones, however, no effect on lipid profile was observed in postmenopausal women with hypercholesterolemia [161]. In another study with postmenopausal hypercholesteremic participants, after a 6-week daily intake of 90 mg of isoflavones, vascular reactivity was improved, but blood cholesterol was not lowered [162]. A recent meta-analysis determined that soy isoflavones significantly reduced serum total and LDL cholesterol but had no influence on HDL cholesterol. The extent of LDL level reduction was greater in participants with hypercholesterolemia than in those without hypercholesterolemia [163]. 

Although several isoflavones function as PPARγ agonists, their intake does not cause weight gain as has been described for full agonists like glitazones. In fact, in various animal and human studies, isoflavone intake has led to a slight weight reduction [133,152,164,165,166]. 

The anti-inflammatory activity of isoflavone supplementation was also demonstrated in several human and animal studies. In animal models, soy isoflavones reduced LPS-induced inflammation by reducing IL-1β, IL-6, NO, and PGE2 production [167]. In hyperlipidemic rabbits, the level of C-reactive protein (CRP) was reduced [158]. Soy isoflavone intake has led to a significant reduction of blood CRP, IL-6, and TNFα levels in a study of patients with end-stage renal failure and systemic inflammation [168]. Conclusively, isoflavones exert simultaneous anti-inflammatory and antilipidemic activity, thus putatively leading to more effective agents for preventing or reducing atherosclerosis.

The anti-inflammatory activity of isoflavones not only improves atherosclerosis but also helps with other diseases associated with inflammation. Examples are the improvement of chronic colitis in a rodent model [169], inhibition of LPS-induced dopaminergic neurodegeneration in rats [143], amelioration of collagen-induced rheumatoid arthritis in a rodent model [170,171], inhibition of pro-inflammatory cytokines in a neurodegenerative cell system [137], reduction of airway inflammation in an *in vitro* system due to inhibition of eosinophil leukotriene synthesis [172], amelioration of alveolitis [145], and putative prevention of osteoporosis due to anti-inflammatory activity in osteoblasts [140].

Of great importance is the physiological relevance of *in vitro* data. The serum concentration of isoflavones in humans after administration of supplements of concentrated isoflavones can reach approximately 10 µM [173]. An isoflavone-rich diet leads to plasma concentrations of 1 to 2.4 µM [174]. Those are ranges in which isoflavones already exert their PPAR activation or anti-inflammatory activities.

### 1.5. Xenobiotic Metabolism and Cell Cycle Control

Isoflavones are known as multitasking bioactive compounds. Their best-investigated aspect is their (anti)estrogenic activity. But as described above, they also modulate PPAR signal cascades. Beyond that, these compounds are ligands of the aryl hydrocarbon receptor (AhR). In the following section, we will describe this receptor and its implications in physiological processes, as well as possible effects of isoflavones via AhR activation.

#### 1.5.1. The aryl hydrocarbon receptor

The AhR is a transcription factor involved in developmental processes as well as in normal physiological pathways such as cell cycle regulation or xenobiotic metabolism. It is a member of the basic helix-loop-helix (bHLH) Per-ARNT-Sim (Pas) family and also shares elementary features of the mode of action of nuclear receptors. Reports have clearly established manifold crosstalk and interaction with nuclear receptors [175,176,177]. The AhR is a phylogenetically ancient protein that has been conserved during evolution [178] because of its important adaptive functions regarding extrinsic signals, such as light and exogenous compounds as well as metabolism and cell cycle control. These functions are also reflected in the diversity and heterogenicity of its ligands, which include physiologically occurring compounds like tryptophan [179], arachidonic acid metabolites [180,181], heme metabolites [182], indigoids [183,184], cAMP [185], equine estrogen [186], and UV products of tryptophan [187]; plant-derived compounds such as indoles [179,188,189] and flavonoids [190,191]; and anthropogenic chemicals such as dioxin [192], polybrominated diephenyl ethers [193], and polychlorinated biphenyls [194]. Beyond that, it is believed that the AhR has endogenous ligands that have not been found so far, although it has been intensively studied since its discovery in 1976 by Poland *et al.* [195]. Furthermore, its expression patterns during embryonic stages indicate a significance of this receptor in development and ontology that is very likely not driven by exogenous ligand activation. Studies with AhR knockout mice have shown severe impairment of organ functions including liver, immune system, and reproductive organs because of deficient differentiation processes arising from lost AhR functions. 

Given the role of AhR in mediating adaptation responses to environmental signals, important AhR target genes include those of the xenobiotic signal transduction pathway, such as those encoding enzymes of phase I and II of xenobiotic metabolism like *CYP1A1* and *GSTYa*. But as would be expected from its functions in cell regulation and apoptosis, this receptor also controls genes encoding regulators of growth, cell proliferation, and the cell cycle. 

The entirety of AhR functions that are mediated via isoflavones through agonistic or antagonistic modulation of this pathway remains elusive. Nevertheless, isoflavones can be regarded as selective AhR modulators (sAhRMs). 

#### 1.5.2. AhR *in vitro* assays

Given the heterogenicity and variety of AhR ligands [179,180,181,182,183,184,185,186,193,194,196,197,198,199,200,201,202,203,204,205,206,207,208,209], using easily executed screening assays to identify its ligands only makes sense. Several *in vitro* test systems that screen for AhR ligands have been reported. First and foremost, these screenings have been implemented as operative instruments in the search for endocrine disrupters, as it has been shown that pollutants can exert anti-estrogenic effects via AhR that include a modulation of ER pathways without direct interaction with the ERs [210,211,212,213,214]. Because of this background and the high affinity of anthropogenic halogenated aromatic hydrocarbons (HAHs) for the AhR, a chemical class that includes polychlorinated biphenyls (PCBs) and polychlorinated dibenzodioxins (PCDDs), but also non-halogenated polycyclic aromatic hydrocarbons (PAHs) [194,207,208,209], toxicologists have intensively studied the AhR for a long time. Over the years, the research focus has shifted towards naturally occurring AhR ligands that could act as sAhRMs and could be useful in cancer prevention and therapy [215,216]. Because a wide spectrum of flavonoids that occur abundantly in medicinal plants as well as in food function as AhR ligands [189,191,217,218,219,220,221,222,223], the elucidation of AhR activation via those compounds has become of great interest. 

**Table 3:** Agonistic and antagonistic effects of isoflavones on the AhR.

Agonistic effects	Antagonistic effects	Assay	Ref.
Dai(+)*	Dai(-), Gen(-)	Gel mobility shift assay (agonistic effects)	[220]
		LBA (rat hepatic cytosol) (antagonistic effects)	
	Dai(-), Gen(+), Gly(-), Equ(+)	LBA (mammalian liver cell cytosol)	[218]
Dai(+), Gen(+), Gly(+), Equ(-)		CALUX (mouse hepatoma cells)	[217]
	Gen(-)	LBA (rat hepatic cytosol)	[224]
	Dai(+)*,Gen (-) Dai(-),Gen (-)	SW-ELISA (Hepa-1c1c7)	[225]
	Dai(+)*,Gen (-) Dai(-),Gen (-)	CALUX (HepG2 cells)	
Dai(+), Gen(+)		Transactivation assay (Hepa-1 cells)	[190]
Dai(-), Gen(-)		Transactivation assay (HepG2 cells)	
Dai(-), Gen(-)		Transactivation assay (MCF-7 cells)	
	Dai(-), Gen(-)	LBA (rat hepatic cytosol)	[191]
Dai(+)*, Gen(+)*	Dai(+), Gen(+)	CYP1A1 expression in HepG2 cells	[226]
Bio(+)	Bio(+)	CYP1A1 expression in MCF-7 cells	[227]
		LBA (rat hepatic cytosol)	
Bio(+)*	Bio(+)	CALUX (MCF-7 cells)	[228]
		CYP1A1 and CYP1B1 expression in MCF-7 cells	
Bio(+)^#^, Dai(-), Equ(+)*, For(+)^#^, Gen(-)		Transactivation assay (yeast)	[189]

Biochanin A (Bio), Daidzein (Dai), Equol (Equ), Formononetin (For), Genistein (Gen), Glycitein (Gly), (+) effect, (-) no effect, * weak ligand, # potent activator, ligand binding assay (LBA), HepG2 (human hepatocellular carcinoma cell line), Hepa-1 (murine hepatoma cell line), MCF-7 (human breast cancer cell line).

*In vitro* bioassays can be used to examine whether a compound can induce (a) AhR transformation, nuclear accumulation, and DNA binding as measured by gel retardation analysis, (b) displacement of labeled AhR ligands in competitive ligand binding assays, or (c) expression of target genes or enzyme induction. Examples of applied assays are listed in Table 3. Some of the assays allow a distinction between agonist and antagonists. The chemically activated luciferase expression assay is a transactivation assay that has been used to measure whether a compound can induce AhR-dependent gene expression in intact cells. Similar test systems based on yeasts as model organisms rather than mammalian cells as well as other reporter systems (e.g., β-galactosidase instead of luciferase) have been used. Cell lines with endogenous receptor expression can be used for the measurement of endogenous target gene expression. These tests are more complex and time-consuming but also provide more specific information. 

Overall, in various *in vitro* bioassays, isoflavones exhibit agonistic or antagonistic effects on the AhR, as summarized in Table 3.

Depending on test systems, small discrepancies among the results exist. Daidzein and genistein seem to be only weak agonists or partial agonists [220,226], while biochanin A and formononetin have exhibited potent agonistic properties in a recombinant yeast transactivation assay [189]. Chan *et al.* [228] found biochanin A to be only a weak AhR agonist. The reasons for the inconsistency of results are explained by different cell lineages as well as the origin of the AhR. Generally, it is recommended that assays should involve human AhR in recombinant systems because species differences in sensitivity have been observed [229]. Also, there is the consideration that most assays are performed with mammalian cell lines, which contain more metabolizing enzymes than yeast. Metabolism via hepatic cells could lead to different results because the compound that elicits the measured effect could be the metabolite and not the parent compound. On the other hand, these results are expected to be a better reflection of the real *in vivo* situation.

#### 1.5.3. Cytochrome P450 enzyme CYP1A1

Organisms are exposed to a multitude of compounds through environment and food. Whether the exposure is volitional or not, eventually most of these compounds must be eliminated in one form or another from the body. To cope with the elimination of endogenous or exogenous compounds, the organism has a detoxification system that includes various enzymes. During phase I of xenobiotic metabolism, compounds are oxidized with the objective of achieving higher polarity and reactivity in preparation for the conjugation reaction of phase II, which leads to production of more hydrophilic compounds. Phase I reactions are accomplished mostly by cytochrome P450 enzymes that catalyze monooxygenase reactions. Among others, the enzymes CYP1A1, CYP1A2, CYP1B1, and CYP2S1 are classical target genes of the AhR [230,231,232]. Toxicologists have intensively studied CYP1A1 because it is responsible for the bioactivation of several carcinogenic compounds. The current general view on the impact of CYP1A1 has been undergoing a change, however. Some compounds cannot be detoxified without a preceding CYP1A1 activation and the aftermath without CYP1A1 is much more severe, which appears to contradict the fact that this same enzyme is responsible for bioactivation pathways producing noxious metabolites. Although CYP1A1 knockout mice are viable, develop normally, and show no obvious difference in phenotype compared to wild-type littermates [233], they die within 30 days after benzo[a]pyrene exposure while wild-type mice show no outward signs of toxicity [234]. 

Thus, a total blockade of CYP1A1 is not advisable because it is indeed part of the detoxification system. The crucial factor is a balanced action of phase I and phase II enzymes. Nevertheless, a modulation of this pathway as a whole, instead of a targeted knockdown of one enzyme, could be useful. Also potentially useful would be knowledge of exactly how the modulation occurs, considering that the composition of ingested food could interfere with administered therapeutics. An example is grapefruit juice, which alters the pharmacokinetics of several drugs via interaction with CYP3A4 (as reviewed by Nowack [235]). 

Many naturally occurring plant compounds interact with the xenobiotic pathway, functioning as AhR ligands, including isoflavones. Their modulation of CYP1A1 can take place in various ways, as will be discussed in the following. Most studies report a suppression of AhR-agonist–induced CYP1A1 expression [236,237,238,239,240,241]. It is not quite clear to what extent this effect is caused by AhR-antagonistic abilities of the isoflavones or if other bioactive properties of these compounds are responsible. Backlund *et al.* [236] reported for genistein and daidzein an inhibition of omeprazole-induced CYP1A1 expression but not for the CYP1A1 expression mediated by 2,3,7,8-tetrachlorodibenzo-p-dioxin (TCDD) and benzo[a]pyrene. Moreover, genistein potentiated induction caused by TCDD. Daidzein, on the other hand, inhibited omeprazole-stimulated CYP1A1 gene transcription but not complex formation of the AhR with its xenobiotic response elements, mediated by omeprazole. Also, daidzein did not inhibit TCDD-mediated CYP1A1 induction at the enzyme, mRNA, and transcriptional levels. The different modes of action may arise from the fact that genistein is a tyrosine kinase inhibitor. Lemaire *et al.* [241] investigated this question experimentally and found that another tyrosine kinase inhibitor inhibited CYP1A1 induction caused by omeprazole. In that study, genistein could not inhibit omeprazole-induced CYP1A1 expression, but the authors concluded that the failure was the result of a lower genistein concentration that was used because of the sensitivity of the cell model. As noted in earlier sections, isoflavones have been described as agonists as well as antagonists of the AhR. Thus, it is not surprising that studies report a direct induction of CYP1A1 expression mediated by isoflavones [226,227,228,237], while other studies did not report such results [242,243].

Isoflavones act also at a non-transcriptional level and directly inhibit the enzyme activity of CYP1A1 [226,228,237,244,245,246,247]. The inhibited metabolism of various compounds could account for the chemopreventive effects of isoflavones. 

Whether or not the CYP1A1-modulating effects of isoflavones are beneficial will depend not only on concerted action with other enzymes of the xenobiotic pathway but also on cell type or content. For example, CYP1A1 expression differs in human breast epithelial cells and breast tumor cells. While non–tumor-derived cells express intermediate CYP1A1 mRNA levels, ERα-positive tumor cells express high levels, and CYP1A1 mRNA expression in ER-negative tumor cells is minimal or negligible  [248]. 

#### 1.5.4. Cell cycle control

The control of the cell cycle is one of the principal tasks of the cell. Although the process is routine, the cell makes a decision at every nanosecond about its fate that can compromise normal replication, apoptosis, necrosis, or uncontrolled growth that can finally lead to cancer development. The AhR is known to regulate cell cycle progression through the control of several cell cycle checkpoint regulators. AhR ligands can arrest cells in various cycle phases. Examples of AhR-regulated cell regulators are Akt, p21, p27, p53, Bax, RelB, and NFκB  [249,250,251,252,253,254]. Among others, these proteins cause cell growth inhibition through arrest or lead cells toward apoptosis. 

Normally, Akt triggers survival signals in cells and functions as an anti-apoptotic factor. Because deregulated Akt signaling is associated with tumor promotion, the downregulation of Akt could be a target in cancer therapy. AhR-deficient cells show impairment in the Akt pathway, leading to the postulation that AhR antagonists could be useful as agents in cancer therapy [250]. A dysregulated NFκB cascade has also been associated with tumor promotion and inflammation. Patel *et al.* [255] reported the suppression of NFκB target gene expression arising from AhR activation by ligands, although the data indicated that no AhR target gene transcription was involved in this process. The antiproliferative effects of an agonist-activated AhR pathway are also mediated via the induction of tumor suppressors or the pro-apoptotic proteins p21, p27, p53, and Bax [249,252,253,254].

Several reports have shown the cell cycle–arresting effects of isoflavones. Given that the isoflavones act not only through the AhR pathway, it is not quite clear to what extent these effects are mediated via the AhR. Nevertheless, the effects obviously can be attributed at least partly to the AhR cascade. The ER pathway seems unlikely to be a mediator of the cell cycle–arresting effects of isoflavones, given that estrogens instead are assoicated with cell cycle promotion according to their physiological role in normal tissue proliferation. This association is true not only for tissues that are known to depend on the ER pathway for proliferation such as the breast, but also for others such as the urinary system [256]. 

Because isoflavones are also known PPAR ligands, this route would also be a possibility for their cell cycle–interfering abilities. The natural PPARγ ligand, 15d-PGJ_2_, a prostaglandin, represses cyclin D1 and inhibits cells in G1/S transition in a PPARγ-pathway–dependent manner [257]. 

As Table 4 shows, most studies have focused on genistein, and only a few reports have involved daidzein or other isoflavones. Also, it is evident that genistein causes an arrest in the G2/M phase of the cell cycle, while it seems that daidzein arrests cells in G0/G1. Concomitant with this arrest, several tumor suppressors are induced and key proteins modulated. Some studies have also reported tumor growth reduction in xenograft models or induction of apoptosis.

**Table 4:** Effect of isoflavones on the cell cycle in human cells.

Effect on cell cycle(cell type)	Further effects	Tested isoflavone (concentration)	Ref.
G2/M arrest(colon cancer)^a^		Genistein (111 µM)	[258]
G2/M arrest(prostate cancer)^b^	Concomitant decrease of cyclin B	Isoflavones from soybean cake; genistein most efficient (30–50 µM)	[259]
G2/M arrest(bladder cancer)^c^	Inhibition of cdc2 kinase activity	Genistein (37 or 185 µM)	[260]
	Direct induction of apoptosis without alteration of cell cycle distribution	Daidzein (39.3 or 196.7 µM) and biochanin A (35.2 or 175.9 µM)	
	Suppression of tumor growth *in vivo* (xenograft model; mice)	Genistein and combined isoflavones	
G2/M arrest(prostate cancer)^d^		Genistein (18.5–74 µM)	[261]
G2/M arrest(breast cancer cells overexpressing Bcl-2)^e1^		Genistein (50 µM)	[262]
G0/G1 arrest(control breast cancer cells)^e2^		Genistein (50 µM)	
G2/M arrest(bladder cancer)^f^	Reduction of tumor volume *in vivo* (xenograft model; mice)	Genistein (50 µM)	[263]
G2/M arrest(androgen-insensitive prostate cancer)^g1^	Induction of tumor suppressor gene expression (p21, p16)	Genistein (10 or 25 µM)	[264]
G0/G1 arrest(androgen-sensitive prostate cancer)^g2^	Induction of apoptosis(only in androgen-insensitive cells)	Genistein (10 or 25 µM)	
G2/M arrest(liver cancer)^h^	Induction of tumor suppressor genes expression (p21),Accumulation of p53 protein	Genistein (37–111 µM)	[265]
G2/M arrest(leukemia cells)^i^	Stimulates Raf-1 activation, Decreases Akt activation, Induction of p21 and cyclin B expression, Induction of apoptosis	Genistein (10 or 25 µM)	[266]
G2/M arrest(prostate cancer)^j^	Increased p21 expression, Decreased cyclin B expression, Decreased NFκB activity	Genistein (15 or 30 µM)	[267]
G1 cell arrest(androgen-sensitive prostate cancer)^k^	Increased p27 and p21 expression	Genistein (≤20 µM)	[268]
	Induction of apoptosis	Genistein (40–80 µM)	
G2/M arrest(non-tumorigenic breast cells)^l^	Enhanced expression of p21 and p53, but not p27	Genistein (30 µM)	[269]
G2/M arrest(prostate cancer)^m^		Genistein (20–100 µM)	[270]
G2/M arrest(B cell leukemia)^n^	Decreased IL-10 secretion, Upregulation of IFNγ	Genistein (7.5–60 µM)	[271]
G2/M arrest(breast cancer)^o^	Increased cyclin B	Genistein (15 or 30 µM)	[272]
G2/M arrest(eye cancer; choroidal melanoma)^p^	Induction of p21, but not required for cell cycle arrest	Genistein (30 or 60 µM)	[273]
G2/M arrest(eye cancer; choroidal melanoma)^q^	Upregulation of CDK1 and p21, but no effect of CDK2 and p27	Genistein (30 µM)	[274]
G1 cell arrest(eye cancer; choroidal melanoma)^q^	Upregulation of CDK2 and weakly p21 and p27	Daidzein (150 µM)	
G2/M arrest(eye cancer; choroidalmelanoma)^r^	Impairment of CDK1 dephosphorylation, Weak accumulation of p53 protein	Genistein (60 µM)	[275]
G2/M arrest(metastatic melanoma)^s^		Genistein (60 µM)	[276]
G2/M arrest(gastric cancer)^t^		Genistein (25 or 60 µM)	[277]
G1 cell arrest(gastric cancer)^t^		Daidzein (25 or 60 µM)	
G2/M arrest(metastatic melanoma)^u^		Genistein (60 µM)	[278]
S phase arrest(metastatic melanoma)^u^		Daidzein (60 µM)	
G0/G1 arrest(colon cancer)^v^	Biphasic effect on cell growth	Daidzein (5–100 µM)	[279]

Listing of cell lines: **a**: Caco2-BBe, **b**: LNCap and PC-3, **c**: RT-4, J82, HT-1376, T24, TSGH8301, BFTC905 and E6,**d**: PC-3, **e1**: MCF-7/PV, **e2**: MCF-7/Bcl-2, **f**: HT-1376, UM-UC-3, RT-4, J82, and TCCSUP, **g1**: DuPro, **g2**: LNCap, **h**: HepG2, **i**: HL60 and NB4, **j**: PC-3, **k**: LNCap, **l**: MCF-10F, **m**: DU-145, **n**: Raji, 2F7 and JVM-13, **o**: T47D, ZR75.1, MDAMB-231 and BT20, **p**: OCM-1, **q**: OCM-1, **r**: OCM-1, **s**: UISO-MEL-6, UISO-MEL-4, UISO MEL-7 and UISO-MEL-8, **t**: HGC-27, **u**: WM451, **v**: LoVo.

#### 1.5.5. AhR activation by isoflavones and health effects

In addition to a role in prenatal development and organogenesis, the AhR is in charge of several housekeeping functions. In normal physiology, this transcription factor regulates the cell cycle, metabolism, and reproduction. Transcriptomic analysis of tissue from AhR knockout mice has revealed that the AhR also regulates genes involved in protein synthesis, tissue maintenance, cell growth, differentiation, and apoptosis [280]. Gene expression profiling by Yoon *et al.* [281] extended the AhR sphere of influence to chemotaxis, immune response, signal transduction, inflammation, and tumor suppression. An activated AhR mediates all these functions. Because isoflavones act as selective AhR modulators, they are putative activators of the abovementioned AhR functions.

The AhR has been intensively studied by toxicologists, because of TCDD-induced toxic responses. In the meantime, it emerged that those effects are mediated by a deregulated or over-activated AhR pathway resulting in a homeostatic imbalance (reviewed by Bock *et al.* [282]). TCDD has a half-life of several years in humans [283,284]. Due to its poor metabolism, TCDD activates the AhR cascade constitutively and elicits toxic responses such as impaired liver regeneration [285], the development of several tumor types [286,287,288] and inflammatory skin lesions [289] have been reported. Several studies evaluated the antagonistic properties of naturally occurring plant compounds on the AhR and the possibility to antagonize TCDD effects [191,203,218,220,221,222,225]. 

But beside a constitutive activation of the AhR signalling cascade, the activated AhR can lead to the bioactivation of compounds during the xenobiotic metabolism. But as we have discussed in a previous chapter, a detoxification without a preceding CYP1A1 activation is even more problematic. It is noteworthy to mention that an activation of the AhR and the induction of CYP1A1 is not synonymous with toxic effects. Several AhR agonists are FDA-approved marketed therapeutics and are not toxic to rodents or humans [290]. 

Nevertheless, possible negative aspects mediated by AhR activation can not be excluded. This could be also true for isoflavones, especially when the intake is extremely high due to excessive recommendations in package inserts of some dietary supplement products. Recommendations that are based on the intake of isoflavones by Asians, will probably not exert harmful effects. 

The AhR functions as a master regulator of several other cell cycle regulators. Among others, the AhR leads cells towards apoptosis by regulation or interaction with Akt, NFκB, RelB, p21, p27, p53, and Bax. As described above, all of these proteins have influence on cell fate and can shift the balance to apoptosis when they are upregulated or downregulated, respectively. 

Studies have reported the same effects for the isoflavones (see also Table 4). Because they are bioactive compounds that stimulate more than the AhR cascade, it is not quite clear which of these effects can be attributed solely to AhR activation. It is only of theoretical interest, however, to separate the AhR-mediated isoflavone actions because *in vivo*, the sum of all effects will always be displayed. 

The anticarcinogenic properties that have been attributed to isoflavones arise in all likelihood from the concerted action that is partly the result of AhR modulation and manifests in a) cell cycle regulation, b) chemoprevention due to CYP enzyme activation, c) antiproliferative and apoptotic effects mediated by up- or downregulation of tumor suppressors or promotors, d) anti-estrogenicity that is a result of the AhR/ER interaction, and e) anti-inflammatory responses. 

## 2. General Conclusion

Certain effects of isoflavones are mediated by either the PPARs or the AhR. With the analysis of *in vitro* effects it is possible to assign them to a mode of action and the associated receptor that mediates those effects. This is a methodical approach to dissect isoflavone action for a better understanding. Methodological shortcoming of in vitro studies is often the use of high isoflavone concentrations, which limits interpretation of the results and makes a comparison with in vivo data difficult.

From the receptor interaction it is clear that isoflavones have an effect on the blood lipid profile, which is explained by the activation of PPAR pathways. This may also counteract certain symptoms of the metabolic syndrome. Isoflavones have also been suggested for prevention of the polycystic ovary syndrome.

Its action on cancer may be partially due to an activation of the AhR pathway and the interaction of the AhR with the ER. Both effects have also been seen *in vivo* in clinical trials. Effects *in vivo* are modulated by bioavailability, which can limit the uptake of bioactive compounds to a great extent, but also metabolism to probably more or less active compounds. This also explains the inter-individually response to isoflavones.

Isoflavones are one of the best studied class compounds, but the focus was primarily on estrogenicity and other effects were mostly overlooked.
